# Adjuvant therapies by anthocyanins potentially mitigating drug resistance and metastatic disease risks in stratified breast cancers: the 3PM-conceptual innovation targeted to NF-kappaB/EMT plasticity

**DOI:** 10.1007/s13167-026-00463-6

**Published:** 2026-07-13

**Authors:** Anita Sventekova, Peter Kubatka, Iva Slaninova, Daniela Nykodymova, Lubica Hornakova, Alexandra Trbolova, Vadim Goncharenko, Karel Smejkal, Olga Golubnitschaja

**Affiliations:** 1 Small Animal Clinic, University of Veterinary Medicine and Pharmacy, Kosice, 041 81 Slovakia; 2 Department of Natural Drugs, Faculty of Pharmacy, Masaryk University, Brno, 625 00 Czech Republic; 3 European Association for Predictive, Preventive and Personalized Medicine (EPMA), Brussels, 1160 Belgium; 4 Department of Biology, Faculty of Medicine, Masaryk University, Brno, 625 00 Czech Republic; 5 Department of Molecular Pharmacy, Faculty of Pharmacy, Masaryk University, Brno, 625 00 Czech Republic; 6 The Women’s Health Centre, Feofaniya Clinical Hospital of the State Management of Affairs of Ukraine, 21 Akademika Zabolotnoho St., Kyiv, Ukraine; 7 Predictive, Preventive and Personalized (3P) Medicine, Department of Radiation Oncology, University Hospital Bonn, Rheinische Friedrich-Wilhelms-Universität Bonn, 53127 Bonn, Germany

**Keywords:** Predictive preventive personalized medicine (PPPM / 3PM), Anthocyanins, Anthocyanidins, Therapy-resistant breast cancer, Triple-negative breast cancer, Trastuzumab resistance, Taxane resistance, NF-κB, Epithelial–mesenchymal transition, Drug sensitization, Mitochondrial homeostasis and biosensorics, Patient stratification, Chronic inflammation, Sympathetic overdrive phenotype

## Abstract

Therapy resistance in breast cancer is increasingly understood as a dynamic state transition driven by inflammatory survival signaling, phenotypic plasticity, and treatment-induced adaptive rewiring rather than as a single endpoint event. Although anthocyanins and anthocyanidins are widely discussed as anticancer phytochemicals, their relevance to the therapy resistance depends on their capacity to modify defined therapy-resistant states under pharmacologically meaningful conditions. Contextually, this article critically evaluates the preclinical evidence and proposes the 3PM-guided conceptual innovation for anthocyanin- and anthocyanidin-linked drug sensitization in therapy-resistant breast cancers, using specifically the NF-κB/EMT crosstalk as a functional lens. To this end, NF-κB/EMT signaling as the proposed target, per strong scientific evidence, is involved in pathways considered central for the phenotype-specific epigenetic regulation of health risks, malignant transformation, development of metastatic disease and therapy resistance. Finally, patient phenotyping and stratification, targeted risk mitigation and treatments tailored to individualized patient profiles are considered essential pillars for improved individual outcomes in the 3PM-guided paradigm.

**Graphical Abstract:**

**Figure:**
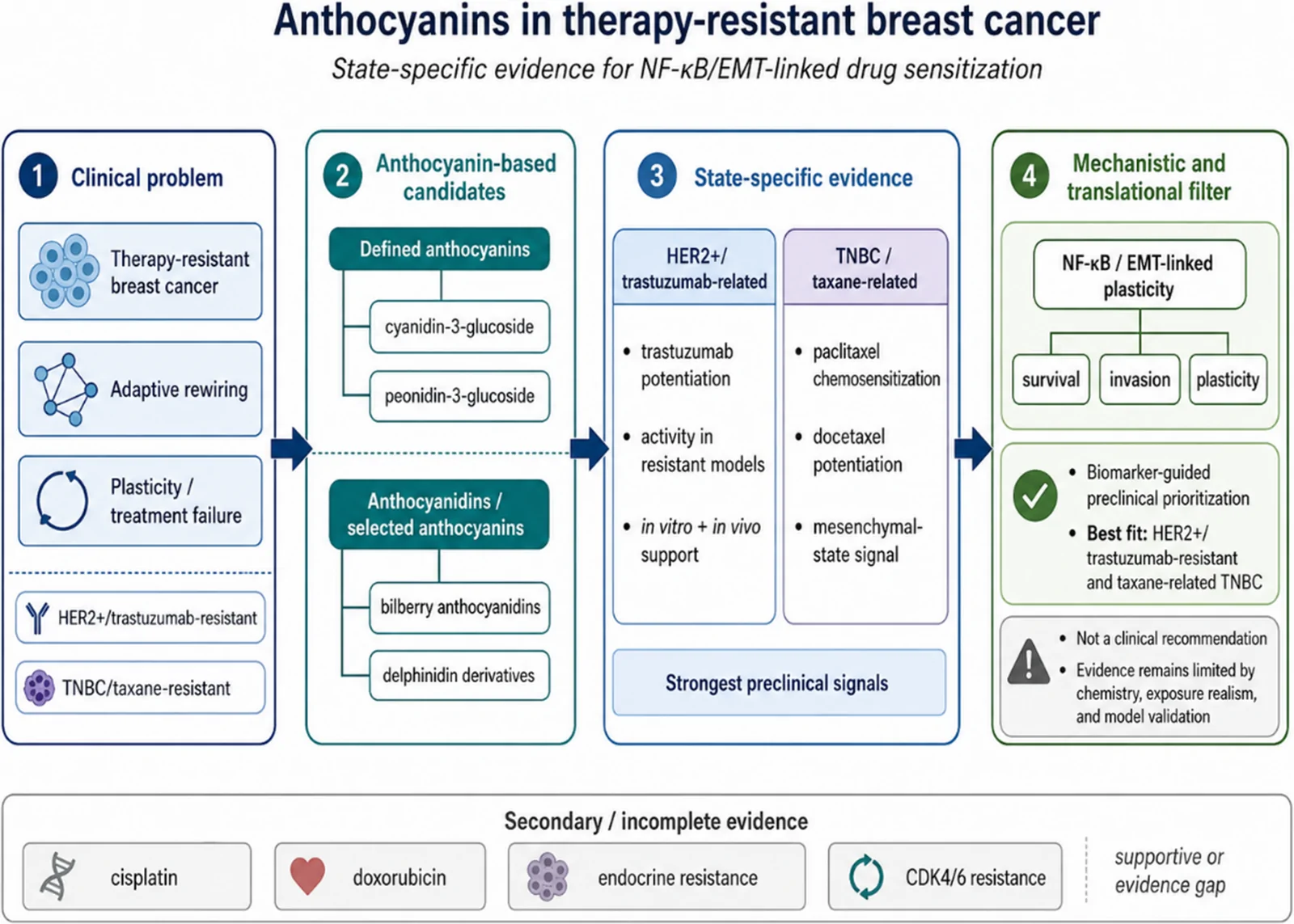

## Introduction

### Therapy resistance as the central clinical problem in breast cancer

The central clinical problem in breast cancer is not the existence of a single aggressive phenotype, but the ability of tumor cells to adapt under treatment pressure. Durable failure emerges when initially responsive disease shifts into resistant states sustained by phenotypic plasticity, inflammatory signaling, stem-like survival programs, and adaptive rewiring, rather than by a single fixed lesion [1–5]. In practical terms, endocrine resistance, HER2-directed resistance, and chemotherapy refractoriness are better understood as state transitions supported by context-dependent survival circuitry than as isolated endpoint events. Therapeutic failure is therefore approached here as a problem of dynamic tumor remodeling and altered response behavior. The discussion is restricted to therapy-resistant states rather than breast cancer as a broadly defined disease category.

### Why anthocyanins are examined here as sensitization candidates rather than as generic nutraceuticals

Anthocyanins have already been widely reviewed as antioxidant, anti-inflammatory, anti-invasive, pro-apoptotic, and chemopreventive phytochemicals in breast cancer and beyond [6]. Repeating that literature would add volume but not precision. The more useful question is narrower: do anthocyanins produce credible drug-sensitizing effects in defined therapy-resistant breast cancer states? The PubMed-indexed evidence suggests that the strongest signal does not come from generic growth-inhibition studies, but from a smaller subset of reports using therapy-relevant models, including combinations with trastuzumab in HER2-positive cells, activity in trastuzumab-resistant disease, and chemosensitization of paclitaxel-resistant TNBC [7–9]. By contrast, many other anthocyanin studies in breast cancer remain mechanistically interesting but do not yet qualify as true resensitization evidence because they lack a resistant-state comparator, an active therapeutic partner, or both. Accordingly, anthocyanins are treated here as conditional sensitization candidates whose relevance depends on the resistant state, pathway context, and strength of the supporting evidence.

### Why NF-κB/EMT targeted conceptual innovation

NF-κB and EMT are in focus of the presented 3PM-based conceptual innovation, because together they capture a clinically meaningful bridge between inflammatory adaptation and phenotypic escape. NF-κB is not simply another signaling pathway in breast cancer; it is a resistance-associated transcriptional hub linked to survival, cytokine amplification, treatment tolerance, and pro-metastatic remodeling [2, 3]. EMT, in turn, is not only a migration program; in breast cancer, it is closely tied to stem-like behavior, altered cellular identity, invasion, dormancy-related fitness, and reduced treatment responsiveness [1, 2, 5]. Most importantly, these axes intersect. Inflammatory tumor-cell plasticity in breast cancer repeatedly converges on NF-κB-centered signaling, while resistant HER2-positive and TNBC states often display EMT enrichment or mesenchymal drift [2, 3, 5, 10]. This point is especially relevant in the HER2 setting, where trastuzumab resistance has been shown to induce EMT and promote conversion of HER2(+) PTEN(-) cells toward a more triple-negative-like resistant phenotype, underscoring that resistance may involve not only pathway escape but also identity shift requiring different therapeutic strategies [11, 12]. Anthocyanin studies become more informative when read through this lens: for example, cyanidin-3-glucoside has been reported to attenuate NF-κB while reversing EMT-like features in TNBC cells [10], which does not, by itself, prove drug sensitization but does provide a mechanistic bridge between anthocyanin exposure and a resistance-relevant plasticity program. For this reason, NF-κB/EMT crosstalk is used as an interpretive filter for resistance-linked sensitization evidence.

### Aim, scope, and conceptual novelty

The central question is whether anthocyanins provide credible preclinical, state-specific evidence for NF-κB/EMT-linked drug sensitization in therapy-resistant breast cancer, and under which translational and 3PM boundary conditions such claims remain plausible [7–10, 13, 14]. The novelty lies in the stricter interpretation of the evidence. The highest weight is given to studies in which four elements converge: a defined resistant state, a therapy-relevant comparator or combination, mechanistic linkage to NF-κB/EMT or an adjacent plasticity node, and an endpoint consistent with resensitization rather than generic cytotoxicity. From a 3PM perspective, the relevant task is to identify resistant states, biomarker contexts, and combination settings in which anthocyanin-based interventions deserve further testing, while avoiding indiscriminate extrapolation.

## Methods

### Study design

This study was prepared as a focused narrative framework review. It was not intended to summarize all anticancer effects of anthocyanins in breast cancer. The study asks a narrower question: whether anthocyanins or anthocyanidins show credible preclinical evidence of drug sensitization in defined therapy-resistant breast cancer states.

The analysis was organized by resistant state rather than by fruit source or compound class. HER2-positive/trastuzumab-related disease, taxane-resistant or mesenchymal triple-negative breast cancer, cisplatin- and doxorubicin-related settings, and endocrine/CDK4/6 inhibitor-resistant disease were considered separately. This structure was chosen because a sensitization claim can only be judged when the resistant phenotype, therapy backbone, tested anthocyanin preparation, and mechanistic readouts are interpreted together.

The study logic followed the general principles of SANRA for narrative reviews, especially a defined aim, transparent literature handling, and balanced interpretation of the evidence. SANRA does not require the same level of search reporting as a systematic review, but it does expect a narrative review to make clear where its information comes from and how the literature was handled [15].

### Literature search and selection

A targeted search was performed in PubMed/MEDLINE and was supplemented by manual screening of references from relevant primary papers and reviews. Scopus, Web of Science, CrossRef, and publisher databases were used when needed to verify bibliographic details and recent publications. The search was updated in May 2026.

Search terms combined anthocyanin-related terms with breast cancer, resistance, drug sensitization, and NF-κB/EMT biology. Representative combinations included: “anthocyanin breast cancer”, “anthocyanidin breast cancer”, “cyanidin-3-glucoside breast cancer”, “anthocyanin trastuzumab resistance”, “anthocyanin HER2-positive breast cancer”, “anthocyanidin paclitaxel-resistant triple-negative breast cancer”, “anthocyanin docetaxel TNBC”, “anthocyanin cisplatin breast cancer”, “anthocyanin doxorubicin breast cancer”, “NF-κB therapy resistance breast cancer”, and “EMT therapy resistance breast cancer”.

Priority was given to studies using breast cancer models with a defined resistant or low-sensitivity phenotype, a standard treatment context, and an interpretable response endpoint. Studies reporting only general cytotoxicity, migration, invasion, antioxidant activity, or anti-inflammatory effects without a treatment-resistance context were used only as a supportive background.

### Evidence handling

Studies were considered central when they included most of the following elements: a resistant or low-sensitivity breast cancer model, an anthocyanin or anthocyanidin intervention, a clinically recognizable therapy backbone, a measurable sensitization or resistance-modifying endpoint, and mechanistic readouts related to NF-κB, EMT, HER2/AKT/MAPK signaling, invasion, angiogenesis, drug-efflux biology, or stress adaptation.

The evidence was then interpreted in four practical categories. Direct preclinical sensitization evidence required a resistant or therapy-relevant model, combination testing with a standard therapy, a measurable response shift, and mechanistic support. Intermediate evidence referred to treatment-relevant studies missing one major element, such as validated resistance, in vivo support, formal interaction analysis, or direct NF-κB/EMT validation. Supportive resistance-adjacent evidence included studies affecting invasion, EMT-like behavior, inflammatory signaling, metastasis, angiogenesis, drug metabolism, or stress adaptation without a strict resensitization design. An evidence gap was used for clinically important resistant states in which anthocyanin-specific sensitization data were insufficient.

This classification was used as an interpretive tool, not as a formal risk-of-bias score. Its purpose was to avoid treating general anticancer activity as proof of therapy resensitization.

### Definition of NF-κB/EMT-linked sensitization

NF-κB/EMT-linked sensitization was defined conservatively. Reduced proliferation, migration, or invasion alone was not considered sufficient. NF-κB support required pathway-proximal or coherent downstream evidence, such as p65 activation, IκBα/IKK-axis modulation, NF-κB transcriptional output, or suppression of NF-κB-regulated inflammatory, survival, angiogenic, or metastatic mediators. EMT support required marker- or regulator-level evidence, including epithelial/mesenchymal markers or EMT-associated transcription factors.

The strongest interpretation was reserved for studies in which these mechanistic changes occurred together with improved response to a standard therapy in a resistant or low-sensitivity breast cancer model. When anthocyanins suppressed resistant-cell growth without showing restored drug responsiveness, the finding was described as resistance-state activity rather than definitive resensitization.

### Translational interpretation

The 3PM interpretation was applied only after the preclinical evidence had been classified. Findings were considered relevant to predictive, preventive, and personalized medicine only when they could support future biomarker-guided testing in defined resistant states. No clinical recommendation was inferred from preclinical data.

Anthocyanin-rich foods, crude extracts, enriched fractions, purified anthocyanins, anthocyanidins, and formulation-dependent preparations were treated as different translational categories. This distinction was necessary because chemical identity, metabolism, stability, bioavailability, and achievable exposure strongly influence whether an in vitro sensitization signal can be considered pharmacologically plausible.

## NF-κB and EMT as the functional lens

### NF-κB as a convergence node in therapy-resistant breast cancer

NF-κB is used here as a functional convergence node because it links several resistance-relevant outputs that are otherwise often discussed separately: survival signaling, inflammatory self-reinforcement, anti-apoptotic transcription, and adaptation to therapeutic stress [16, 17]. In breast cancer, this makes NF-κB more useful than a generic “pathway of interest,” since it can integrate cytokine-driven signaling, transcriptional persistence, and pro-survival buffering across distinct resistant states rather than in only one molecular subtype [18]. The practical importance is that NF-κB activity is not merely correlative: NF-κB inhibition has been shown to enhance tamoxifen sensitivity in resistant breast cancer cells [19], tamoxifen-tolerant ER-positive cells display dependence on NF-κB-linked survival circuitry [20], and therapy-resistant HER2-driven disease has also been discussed in relation to NF-κB-centered pro-survival signaling [21]. NF-κB is therefore treated as a mechanistically actionable, resistance-maintaining hub rather than another item in a broad signaling inventory.

### EMT as a resistance-associated plasticity program

EMT is included primarily as a plasticity program, not only as a metastasis program. EMT-associated change can accompany drug tolerance, invasive persistence, stem-like behavior, and recurrence-related fitness in breast cancer [22, 23]. This is especially important in therapy-resistant disease, where reduced treatment responsiveness may reflect not only pathway escape but also a shift in cellular state toward a more adaptable phenotype [11, 24]. Accordingly, EMT in this manuscript is interpreted as a resistance-linked remodeling of identity and behavior, not as a simple synonym for migration.

Just as importantly, EMT should not be framed as a rigid epithelial-versus-mesenchymal binary. Contemporary EMT literature emphasizes partial or hybrid epithelial/mesenchymal states [25, 26], and these intermediate states are highly relevant to cancer because they may preserve survival flexibility, stem-like capacity, and treatment tolerance without requiring a fully mesenchymal endpoint [22, 27]. That matters here because later evidence should not be judged only by whether cells become “fully mesenchymal,” but by whether resistant phenotypes display coherent EMT-linked plasticity features that are plausibly reversible.

### Why NF-κB/EMT crosstalk is more useful here than a broad pathway catalogue

The reason for selecting NF-κB/EMT crosstalk as the main lens is that it captures a biologically coherent transition from inflammatory survival signaling to phenotypic escape. NF-κB is not only associated with resistance; it can also drive EMT-linked programs through regulators such as Snail, Slug, and Twist in breast cancer models [28, 29]. In HER2-positive disease, trastuzumab resistance has been reported to induce EMT-like reprogramming and subtype drift [11], while IL-6/STAT3/NF-κB-associated circuitry has been implicated in EMT-linked resistance behavior in HER2-positive breast cancer [30]. Together, these observations make NF-κB/EMT crosstalk useful for interpreting resensitization evidence, because they connect survival under treatment pressure with phenotypic state change.

Other pathways remain relevant. PI3K/AKT, TGF-β, MAPK, and cancer-stem-cell-related signaling pathways clearly contribute to the biology of resistance [23, 31–33]. However, they are treated here as secondary or supporting nodes unless they directly reinforce NF-κB-dependent survival output, EMT-associated transcriptional change, or both. This selectivity is deliberate. A broad pathway catalogue may appear comprehensive, but it often weakens mechanistic discipline and blurs the distinction between background biology and resistance-linked circuitry. The NF-κB/EMT intersection provides a narrower and therefore more operational framework.

### Operational meaning of “NF-κB/EMT-linked sensitization” in this article

“NF-κB/EMT-linked sensitization” is used here in a strict operational sense. Mechanistic support is considered meaningful when a study includes direct NF-κB-related readouts, such as p65 activation or nuclear localization, IκBα-related changes, or coherent suppression of NF-κB-regulated inflammatory/survival outputs [16, 17], together with EMT-relevant evidence at the marker or regulator level, for example, E-cadherin recovery, reduction of vimentin or N-cadherin, or modulation of EMT-associated transcription factors such as SNAIL, SLUG, TWIST, or ZEB-linked programs [22, 29, 34]. Anti-migratory effects alone are not sufficient, and generic cytotoxicity is not treated as evidence of NF-κB/EMT-linked resensitization.

A second requirement is functional linkage to therapy response. Plasticity-associated readouts such as colony formation, mammosphere behavior, invasion, migration, or stem-like features may strengthen interpretation when they move in parallel with NF-κB/EMT modulation [24, 34], but the strongest support in this manuscript is reserved for combination settings in which an anthocyanin or anthocyanidin improves response to a standard therapy in a resistant or low-sensitivity model while shifting at least part of the NF-κB/EMT axis in a coherent direction. The later sections, therefore, distinguish general anticancer activity, supportive anti-plasticity biology, and therapy-linked sensitization instead of treating these categories as interchangeable.

## State-specific evidence in HER2-positive/trastuzumab-resistant breast cancer

### Why trastuzumab resistance is prioritized

Trastuzumab resistance deserves priority because it connects a clinically established therapeutic axis with one of the clearest preclinical anthocyanin signals currently available in breast cancer sensitization research. HER2-positive breast cancer remains one of the best-defined targetable breast cancer subtypes, and HER2-directed therapy has substantially changed its clinical management [35]. At the same time, intrinsic and acquired resistance to trastuzumab remain major limitations, preventing durable response in a clinically relevant proportion of patients [36, 37].

This resistance state should not be reduced to persistent HER2 expression alone. Trastuzumab failure may involve incomplete HER2 pathway blockade, compensatory receptor tyrosine kinase signaling, PI3K/AKT and MAPK pathway rewiring, PTEN-related escape, altered receptor trafficking, immune-related mechanisms, stem-like adaptation, and broader phenotypic plasticity [37–39]. This matters because anthocyanins should not be assessed as generic cytotoxic phytochemicals in this setting. Their relevance depends on whether they can modify a defined therapy-resistant state in a way that is biologically and pharmacologically meaningful.

The HER2/trastuzumab setting is therefore one of the few anthocyanin-related areas where a state-specific discussion is justified. A natural-product screen identified cyanidin-3-glucoside and peonidin-3-glucoside as selectively active against HER2-positive breast cancer cells [40]. Subsequent work showed that cyanidin-3-glucoside potentiated trastuzumab activity in HER2-positive breast cancer models in vitro and in vivo [7], and a later study directly tested anthocyanins in parental HER2-positive cells and trastuzumab-resistant derivatives [8]. This makes the HER2/trastuzumab section stronger than most anthocyanin literature, which still relies mainly on therapy-naïve growth-inhibition models.

The trastuzumab-resistance focus is therefore justified, although the conclusion remains limited. Current evidence supports further preclinical testing of selected anthocyanins, especially cyanidin-3-glucoside and peonidin-3-glucoside, in HER2-positive/trastuzumab-resistant breast cancer. Clinical recommendation, or a broad claim that anthocyanins overcome trastuzumab resistance in patients, would be premature.

### Direct preclinical evidence for anthocyanin activity in trastuzumab-resistant models

The direct evidence begins with HER2-positive model selection rather than with resistance reversal. In a high-throughput natural-product screen, cyanidin-3-glucoside and peonidin-3-glucoside were identified among compounds with selective anti-proliferative activity against HER2-positive breast cancer cells [40]. This study is best interpreted as a HER2-context entry point, rather than evidence of trastuzumab resensitization. Its value lies in showing that defined anthocyanins can act within a HER2-positive cellular context and influence HER2-associated growth behavior.

A stronger level of evidence comes from therapy-linked combination studies. Cyanidin-3-glucoside was reported to potentiate trastuzumab activity in HER2-positive breast cancer cells, with effects on growth inhibition, apoptosis, HER2, and downstream signaling, and in vivo tumor growth [7]. This study is important because it moves beyond anthocyanin monotherapy and tests whether an anthocyanin can enhance the effect of an established HER2-directed agent. In the framework used here, this represents a credible sensitization signal in a trastuzumab-relevant HER2-positive context.

However, this evidence should not be overstated. The combination data support enhanced trastuzumab activity, but they do not by themselves prove reversal of an established trastuzumab-resistant state. A strict resensitization claim would require a resistant comparator, trastuzumab monotherapy, anthocyanin monotherapy, combination treatment, formal additivity or synergy assessment, and mechanistic confirmation that the resistant phenotype is functionally shifted toward renewed trastuzumab responsiveness [41].

The strongest resistance-state evidence comes from the study that evaluated anthocyanins in parental HER2-positive cells and derivative trastuzumab-resistant models [8]. This study anchors the subsection because it directly addresses trastuzumab-resistant breast cancer biology. Cyanidin-3-glucoside and peonidin-3-glucoside inhibited the growth of trastuzumab-resistant derivatives, suppressed HER2 phosphorylation and downstream AKT/MAPK signaling, induced apoptosis, reduced migration and invasion, and inhibited tumor growth in vivo [8]. These findings provide a legitimate preclinical basis for considering selected anthocyanins as resistance-state modulators in HER2-positive breast cancer.

The interpretation still requires precision. The data support suppression of trastuzumab-resistant HER2-positive phenotypes, but resistant-cell inhibition is not equivalent to proven trastuzumab resensitization. A compound may inhibit trastuzumab-resistant cells through HER2-related or HER2-adjacent mechanisms without restoring trastuzumab dependence. The evidence is therefore best described as direct resistance-state activity with sensitization relevance, not as definitive reversal of trastuzumab resistance [42].

The in vivo evidence strengthens this block because the field often relies on short-term viability assays. Both the HER2-positive combination study and the trastuzumab-resistant anthocyanin study included xenograft-based tumor-growth assessment [7, 8]. Still, xenografts cannot fully reproduce clinical trastuzumab biology, especially immune-mediated mechanisms, clonal heterogeneity, tumor microenvironmental influences, and long-term treatment pressure. The HER2/trastuzumab block is therefore among the strongest preclinical anthocyanin signals in the manuscript, but it remains preclinical.

### NF-κB/EMT relevance in this state: direct, partial, or inferred?

The NF-κB/EMT interpretation of the HER2/trastuzumab block requires careful separation of direct evidence from mechanistic inference. The main anthocyanin studies in this section directly support modulation of HER2 phosphorylation, AKT/MAPK signaling, apoptosis, migration, invasion, and tumor growth [7, 8]. These outputs are highly relevant to trastuzumab-resistant biology, but they do not by themselves establish a complete NF-κB/EMT-linked resensitization mechanism.

NF-κB is relevant in this state mainly as a resistance-associated survival node. Trastuzumab-resistant luminal B breast cancer cells have been reported to acquire basal-like growth features through NF-κB activation, supporting the idea that inflammatory transcriptional rewiring can participate in HER2-targeted therapy escape [43]. This finding fits the logic of the present article, in which therapy resistance is treated as a dynamic state rather than a fixed endpoint. However, the available HER2/trastuzumab anthocyanin studies do not yet show that cyanidin-3-glucoside or peonidin-3-glucoside reverses trastuzumab resistance through direct NF-κB inhibition in the same resistant model. NF-κB is therefore biologically plausible and resistance-relevant in this setting, but it is not yet proven as the direct mediator of anthocyanin-linked trastuzumab resensitization.

The EMT evidence is relevant, but it does not yet close the mechanistic gap. Trastuzumab resistance has been shown to induce EMT and to shift HER2-positive/PTEN-negative breast cancer cells toward a more triple-negative-like resistant phenotype [11]. This is important because it supports the core idea that HER2-directed resistance may involve cellular identity change, not merely incomplete receptor blockade. It also connects the HER2/trastuzumab block to the broader NF-κB/EMT framework of the manuscript.

There is anthocyanin-linked EMT evidence in a HER2-positive context, but it does not fully overlap with trastuzumab resistance. Black rice-derived anthocyanins inhibited EMT-mediated metastatic behavior in HER2-positive MDA-MB-453 cells through suppression of FAK-related signaling, with reported effects on migration, invasion, E-cadherin, vimentin, fibronectin, and FAK/c-Src/p130Cas signaling [44]. This supports the plausibility that anthocyanin-rich preparations can modulate EMT-linked behavior in HER2-positive breast cancer cells. It should not be merged with the trastuzumab-resistant evidence as if it directly proved trastuzumab resensitization, because the study did not test a trastuzumab-resistant model or a trastuzumab-combination setting.

The evidence, therefore, separates into three levels. Direct support exists for anthocyanin activity against HER2-positive and trastuzumab-resistant phenotypes, particularly through HER2 phosphorylation, AKT/MAPK signaling, apoptosis, invasion, migration, and tumor growth control [7, 8]. NF-κB and EMT are strongly relevant to trastuzumab-resistance biology [11, 43], and anthocyanins can affect EMT-linked behavior in HER2-positive cells [44]. What remains missing is direct proof that anthocyanins restore trastuzumab response through a unified NF-κB/EMT mechanism.

### Translational interpretation of the HER2/trastuzumab evidence block

The HER2/trastuzumab section is one of the most clinically anchored parts of the manuscript. In contrast to broad anthocyanin studies in therapy-naïve breast cancer cells, it includes a recognizable disease state, an established therapeutic backbone, defined anthocyanin candidates, combination testing, resistant-model data, and in vivo support [7, 8, 40]. These features give the HER2/trastuzumab evidence a higher weight in a state-specific interpretation.

The strength of this evidence is preclinical, not clinical. It identifies a coherent signal that selected anthocyanins can interact with HER2-positive biology, enhance trastuzumab-relevant activity, and suppress trastuzumab-resistant HER2-positive phenotypes [7, 8]. This supports the manuscript’s central distinction between state-linked sensitization signals and broad, non-specific anticancer claims.

Several limitations still prevent stronger translational conclusions. First, the number of direct HER2/trastuzumab anthocyanin studies is small. Second, the most direct mechanistic evidence is centered on HER2/AKT/MAPK signaling rather than on a fully validated NF-κB/EMT axis. Third, the EMT-relevant black-rice anthocyanin evidence is mechanistically useful but not trastuzumab-resistant or trastuzumab-combination evidence [44]. Fourth, trastuzumab resistance involves immune, signaling, receptor-level, metabolic, and plasticity-related mechanisms that are only partly represented in xenograft systems [37–39]. Fifth, exposure realism remains insufficiently resolved, particularly regarding anthocyanin stability, metabolism, formulation, and achievable tumor concentrations.

In a 3PM-oriented interpretation, this evidence functions as a biomarker-guided preclinical prioritization signal. Future studies should test chemically defined cyanidin-3-glucoside and peonidin-3-glucoside, or standardized anthocyanin mixtures, in validated HER2-positive trastuzumab-resistant models. These models should be stratified by HER2 phosphorylation, PI3K/AKT and MAPK activity, PTEN status, NF-κB activation, EMT markers, invasive behavior, and stem-like features [37]. Combination studies should include trastuzumab monotherapy, anthocyanin monotherapy, combination treatment, formal interaction analysis, and orthogonal validation of NF-κB and EMT if those mechanisms are claimed [41].

Taken together, the HER2/trastuzumab evidence identifies cyanidin-3-glucoside and peonidin-3-glucoside as the most defensible anthocyanin candidates for further testing [7]. The direct support lies in HER2-positive models, trastuzumab-relevant combination data, and activity against trastuzumab-resistant derivatives [8]. NF-κB activation and EMT-like conversion are clearly relevant to trastuzumab resistance, but they have not yet been causally connected to anthocyanin-mediated restoration of trastuzumab response in the same experimental system [11, 43, 44]. The defensible conclusion is limited to biomarker-guided preclinical testing. These compounds cannot yet be described as clinically validated trastuzumab-resensitizing agents.

## State-specific evidence in triple-negative breast cancer: taxane-resistant, mesenchymal, and related settings

### Why TNBC belongs at the center of this manuscript

Triple-negative breast cancer (TNBC) is at the center of this manuscript because it represents a treatment-limited, biologically heterogeneous, and plasticity-rich breast cancer setting in which chemotherapy resistance remains a major determinant of clinical failure. Unlike hormone-receptor-positive or HER2-positive breast cancer, TNBC lacks the classical targets for endocrine or HER2-directed therapy, and anthracycline- and taxane-based chemotherapy remains a central therapeutic backbone, although immune checkpoint inhibitors, PARP inhibitors, and AKT-directed strategies have expanded treatment options in selected molecular contexts [45].

The relevant point is not only TNBC aggressiveness, but its biological heterogeneity. Gene-expression analyses divided TNBC into molecularly distinct subtypes, including basal-like, immunomodulatory, mesenchymal, mesenchymal stem-like, and luminal androgen receptor-enriched forms, later refined into tumor-intrinsic TNBC-type-4 categories with different biological and clinical features [46, 47]. This heterogeneity matters directly for anthocyanin interpretation. Evidence from one TNBC cell model, one extract, or one chemotherapy context cannot be generalized to all TNBC. The key question is therefore whether anthocyanins or anthocyanidins provide credible sensitization signals in defined TNBC states, especially taxane-resistant, mesenchymal, or NF-κB/EMT-enriched settings.

### Paclitaxel-resistant TNBC: anthocyanidins and NF-κB-linked chemosensitization

Taxane resistance in TNBC is multifactorial. It may involve altered microtubule dynamics, drug-efflux transporters, apoptosis evasion, autophagy, cancer-stem-cell enrichment, hypoxia-linked adaptation, and activation of survival pathways such as NF-κB, PI3K/AKT/mTOR, JAK/STAT, and MAPK signaling [48]. For the present argument, the full catalogue of resistance mechanisms is less important than the subset connecting taxane resistance with inflammatory plasticity and anthocyanidin-linked chemosensitization.

The strongest mechanistic bridge is the relationship between mesenchymal phenotype, NF-κB activation, and paclitaxel resistance. Esparza-López et al. showed that mesenchymal primary breast cancer cells were more resistant to paclitaxel than epithelial counterparts, and that paclitaxel resistance was associated with activation of the IKK/IκBα/NF-κB axis. Functionally, blocking NF-κB activation restored paclitaxel sensitivity in resistant mesenchymal cells, supporting NF-κB as a resistance-maintaining node rather than a passive correlate [24]. This finding gives NF-κB/EMT biological relevance as a filter for taxane-resistant TNBC evidence, rather than leaving it as a loose mechanistic label.

The most direct anthocyanidin evidence in this state comes from Aqil et al. The authors tested bilberry-derived anthocyanidins in TNBC models and reported inhibition of TNBC growth and metastasis together with chemosensitization of paclitaxel-resistant TNBC cells through modulation of NF-κB signaling and metastatic/angiogenic mediators [9]. In paclitaxel-resistant MDA-MB-231Tx cells, the anthocyanidin plus paclitaxel combination markedly lowered the paclitaxel IC_50_, and in orthotopic xenografts, the combination reduced tumor volume, whereas either treatment alone was insufficient in the resistant setting [9].

Aqil et al. provide the main Tier 1 TNBC evidence in this study. It contains the elements required by the framework: a resistant TNBC model, a standard chemotherapy backbone, a sensitization endpoint, in vivo support, and a mechanistic linkage to NF-κB-associated invasion, metastasis, and angiogenesis. The conclusion must remain narrow. The data support the use of anthocyanidins as paclitaxel chemosensitizers in a defined preclinical TNBC state. They do not justify a broad clinical claim that dietary anthocyanins overcome TNBC chemoresistance.

### Mesenchymal TNBC and docetaxel potentiation

Mesenchymal TNBC is relevant here because it represents a plasticity-enriched, treatment-resistant-prone state rather than a minor phenotypic variation. EMT-associated features, including reduced epithelial identity, increased mesenchymal-marker expression, enhanced migration, stem-like behavior, and inflammatory survival signaling, can contribute to reduced taxane responsiveness, consistent with evidence linking mesenchymal phenotype, NF-κB activation, and paclitaxel resistance in primary breast cancer cells [24]. The relevant question is whether anthocyanin-related compounds can weaken a taxane-relevant mesenchymal state in a pharmacologically meaningful way, as suggested by docetaxel potentiation data in mesenchymal TNBC cells [49].

Ribeiro et al. recently provided an important taxane-linked signal by testing five structurally distinct anthocyani(di)ns in mesenchymal TNBC cells. Delphinidin-3-O-glucoside and delphinidin-3-O-rutinoside, both trihydroxylated anthocyanins, showed stronger antiproliferative effects than structurally related compounds and potentiated docetaxel activity. The reported combinations allowed substantial docetaxel dose reduction while sparing non-neoplastic mammary cells, suggesting a selective sensitization pattern rather than nonspecific cytotoxic amplification [49].

The study adds two useful points to the TNBC argument. First, it brings chemical structure into the decision framework: anthocyanin activity cannot be discussed as a class effect when trihydroxylation appears to influence taxane potentiation. Second, it extends the taxane argument beyond paclitaxel to docetaxel-relevant mesenchymal TNBC. The evidence still needs careful classification. Ribeiro et al. support docetaxel potentiation in mesenchymal TNBC cells, but they do not yet provide a complete causal demonstration that NF-κB/EMT reversal mediates this effect, nor do they establish reversal of a fully validated docetaxel-resistant derivative. For that reason, the most accurate classification is Tier 2: taxane-relevant, mesenchymal-state-associated, chemically informative, but mechanistically incomplete [49].

Together, Aqil et al. and Ribeiro et al. define the strongest TNBC logic in this manuscript. Aqil et al. provide direct paclitaxel-resistant NF-κB-linked chemosensitization evidence, whereas Ribeiro et al. provide a structurally refined docetaxel potentiation signal in mesenchymal TNBC. This combination justifies TNBC as a central state-specific block without turning the chapter into a general review of TNBC biology.

### Supportive TNBC evidence outside strict taxane-resistant models

Several studies support the broader idea that anthocyanins can modulate TNBC plasticity, invasion, inflammatory signaling, and survival pathways. They are relevant as supportive biology, but they do not provide direct evidence of taxane-resistant TNBC resensitization.

Liang et al. showed that cyanidin-3-glucoside reduced migration and invasion in TNBC cells and promoted a mesenchymal-to-epithelial shift through Sirt1-associated regulation, with changes in EMT markers including E-cadherin, ZO-1, vimentin, N-cadherin, and Snail proteins [10]. Chen et al. similarly reported that cyanidin-3-O-glucoside inhibited EMT, migration, and invasion of breast cancer cells through KLF4 upregulation [50]. These findings fit the NF-κB/EMT-plasticity concept, but because they do not test a resistant taxane state or a taxane-combination design, they should remain supportive rather than central.

Paramanantham et al. provide another useful mechanistic bridge. Anthocyanins from *Vitis coignetiae* suppressed TNF-α-induced NF-κB activation and downstream mediators involved in proliferation, invasion, adhesion, angiogenesis, and metastatic behavior, including COX-2, c-Myc, MMPs, ICAM-1, and VEGF [51]. This supports the plausibility that anthocyanins can attenuate inflammatory pro-invasive signaling, but the model is not equivalent to taxane-resistant TNBC. Therefore, the evidence strengthens the mechanistic background but does not alter the hierarchy of the TNBC block.

The same caution applies to other anthocyanin-rich models. Cyanidin-3-O-glucoside has been reported to inhibit ERα36/EGFR-positive TNBC through EGFR-related signaling and apoptosis [52], while blueberry phytochemicals and whole blueberry powder have shown growth- and metastasis-modulating effects in MDA-MB-231 models through PI3K/AKT and related pathways [53, 54]. These studies broaden the anticancer context, but they do not directly answer the state-specific question of taxane resensitization. Recent in vivo berry-derived evidence further supports this cautious interpretation. In syngeneic 4T1 mouse models, anthocyanin-rich Aronia melanocarpa fruit peel reduced tumor volume by 61%, while bilberry peel-enriched pomace reduced 4T1 tumor volume by up to 91% [55, 56]. These findings strengthen the in vivo plausibility of berry anthocyanin-rich preparations in TNBC-like models, but remain supportive because they do not test taxane resensitization or a defined NF-κB/EMT-linked resistant state.

The recent dark sweet cherry anthocyanin studies are more relevant to resistance biology but still belong outside the core taxane block. Nava-Ochoa et al. reported that anthocyanin-rich cherry phenolics influenced drug metabolism and transporter-related mechanisms in 4T1 breast cancer cells, while additional in vivo work showed suppression of pulmonary metastasis and downregulation of genes linked to metastasis and therapy resistance [57]. A later study connected anthocyanin-rich cherry phenolics to context-dependent modulation of the Nrf2–Keap1–p62 axis in drug-resistant 4T1 cells [58]. These findings preserve the author’s original point that anthocyanin-rich preparations may affect resistance-associated biology beyond taxanes. However, they should be described as adjacent resistance-modifying evidence, not as proof of taxane-specific resensitization.

### What the TNBC evidence does and does not prove

The TNBC evidence supports a focused but limited conclusion. The strongest direct signal comes from paclitaxel-resistant TNBC, where bilberry-derived anthocyanidins chemosensitized resistant cells and xenografts to paclitaxel in association with NF-κB-linked modulation of survival, invasion, angiogenesis, and metastasis-related outputs [9]. A second, although less complete, signal comes from mesenchymal TNBC, where trihydroxylated anthocyanins potentiated docetaxel and introduced a structure-dependent argument for candidate selection [49].

Broader TNBC evidence supports the plausibility of this interpretation. Anthocyanins and anthocyanidins can modulate EMT-like behavior, migration, invasion, NF-κB-related inflammatory signaling, PI3K/AKT or EGFR-linked survival pathways, drug-transporter biology, and metastasis-associated transcriptional programs in TNBC or breast cancer models [10, 50, 51].

These data justify mechanistically focused follow-up studies, but not patient-level recommendations or generalized claims that anthocyanins overcome TNBC chemoresistance. A 3PM-oriented interpretation should therefore focus on biomarker-guided preclinical prioritization in NF-κB-high, EMT-enriched, taxane-resistant, or mesenchymal TNBC states.

Overall, the TNBC evidence is strongest in taxane-related settings. Bilberry-derived anthocyanidins provide the most direct paclitaxel-resistant signal, with NF-κB-linked changes in invasion, angiogenesis, and metastatic behavior. Delphinidin derivatives add a chemically informative docetaxel-potentiation signal in mesenchymal TNBC, although this remains short of validated resistance reversal. Studies involving cyanidin-3-glucoside, Vitis coignetiae anthocyanins, blueberry preparations, and dark sweet cherry phenolics broaden the plasticity and resistance-adjacent background, but most do not test strict taxane-resistant resensitization models. The defensible conclusion is preclinical prioritization in NF-κB-high, EMT-enriched, mesenchymal, or taxane-resistant TNBC.

## Supportive but secondary evidence: cisplatin-related and other non-core settings

### Cisplatin-related low-sensitivity or relatively resistant breast cancer models

Cisplatin-related evidence is relevant, but it does not anchor the study. The available breast cancer studies mostly deal with intrinsically low-sensitivity luminal models, especially MCF-7 cells, rather than with acquired cisplatin-resistant derivatives. This is a useful distinction, because higher cisplatin IC₅₀ values in MCF-7 cells compared with more aggressive breast cancer models may indicate relative intrinsic tolerance, but they do not automatically define a clinically refractory resistant state [59].

The clearest breast cancer example remains the study by Paramanantham et al. [60], which tested anthocyanins isolated from *Vitis coignetiae* Pulliat in MCF-7 cells. The study is useful because it goes beyond a viability curve. Cisplatin alone showed limited activity in this model, whereas the addition of the anthocyanin fraction markedly reduced cell viability and increased the sub-G1 apoptotic fraction. More importantly for the present manuscript, the molecular data linked this effect to suppression of treatment-associated survival signaling: AKT phosphorylation declined, p-NF-κB and p-IκB were reduced, XIAP decreased, and PARP-1 cleavage increased [60].

The interpretation remains limited. These findings indicate that *Vitis coignetiae* anthocyanins can weaken AKT/NF-κB-linked survival adaptation during cisplatin exposure. They do not prove the reversal of acquired cisplatin resistance. No resistant derivative, no long-term selection model, and no formal resistance-reversal design were included. In the logic of this article, Paramanantham et al. [60] therefore provide a useful Tier 2 signal: treatment-linked chemosensitization in a low-sensitivity breast cancer model, with partial mechanistic support through AKT/NF-κB modulation.

Zhao et al. [61] add a more quantitative, but less mechanistically resolved, signal. Using Gardenblue blueberry anthocyanins, they reported a modest reduction in cisplatin IC₅₀ in MCF-7 cells, while anthocyanins alone showed weaker antiproliferative activity. This supports a potentiation effect rather than strong independent cytotoxicity. The limitation is also clear: the molecular analyses were developed mainly outside the breast cancer setting and were centered on apoptosis and ROS-related readouts, without direct NF-κB or EMT interrogation in MCF-7 cells [61]. The study is useful, although its weight in the evidence hierarchy remains limited.

Awad et al. [62] approach the question from a formulation angle. Their folate-targeted carbon nanotube co-delivery system combining cisplatin and anthocyanin nanoparticles improved cytotoxic activity in MCF-7 and HepG2 models and showed apoptosis-associated changes, including Bax/Bcl-2 modulation and reduced TNF-α expression. This is technically interesting, especially because formulation is one of the known translational weaknesses of anthocyanins. Still, the study does not test a resistance-defined breast cancer state, and NF-κB activity is inferred only indirectly through TNF-α-related signaling. Its value is mainly supportive and pharmacotechnological, rather than evidence of state-specific sensitization.

Evidence from non-breast models should remain peripheral. El-Nashar et al. [63], for example, reported a modest reduction in cisplatin IC₅₀ in HeLa cells when cisplatin was combined with pitaya seed extract containing anthocyanidin constituents. The effect was linked to p53-dependent intrinsic apoptosis. For the present article, however, this is background evidence only. It does not involve breast cancer, does not address acquired resistance, and does not strengthen the NF-κB/EMT-specific argument.

Overall, the cisplatin block supports a cautious statement: anthocyanin-containing preparations may enhance cisplatin-associated cytotoxicity in selected low-sensitivity systems, and the strongest breast cancer mechanistic signal points toward AKT/NF-κB suppression. What is missing is just as important: acquired cisplatin-resistant breast cancer models, proper interaction analysis, orthogonal NF-κB/EMT validation, and exposure-realistic dosing. This evidence is best classified as Tier 2. It supports conditional chemosensitization under low-sensitivity conditions, not reversal of established cisplatin resistance.

### Other exploratory combination contexts

Other chemotherapy-combination settings broaden the biological context but do not change the hierarchy of evidence. Among them, the doxorubicin-related studies with dark sweet cherry anthocyanins are the most relevant.

Nava-Ochoa et al. [64] tested dark sweet cherry anthocyanins in a syngeneic 4T1 triple-negative breast cancer model, including a doxorubicin-related treatment context. Anthocyanin intervention was associated with reduced pulmonary metastasis and with downregulation of genes linked to metastasis and therapy resistance. The study also reported effects on markers connected with hypoxia and stemness. At the same time, several inflammatory or EMT-associated mediators were altered across active treatment groups rather than in a clean combination-specific pattern [64]. This makes the paper relevant to treatment-associated plasticity but insufficient as proof of doxorubicin resensitization.

The drug-resistant 4T1-DR model in Nava-Ochoa et al. [58] brings the discussion closer to resistance biology. Anthocyanin-rich dark sweet cherry phenolics modulated the Nrf2–Keap1–p62 axis, with dose-dependent effects on antioxidant-response and autophagy-related readouts. This is relevant because Nrf2-linked stress adaptation and autophagic flux can support survival under drug pressure. The key point, however, is that the resistant phenotype was not convincingly erased. The data suggest interference with stress-response wiring in resistant TNBC cells, not a full reversal of doxorubicin resistance.

Zhao et al. [61] also tested anthocyanins in doxorubicin-related combinations, but the breast cancer relevance is limited. Their stronger combination effects were reported outside breast cancer, whereas in MCF-7 cells, the doxorubicin-combination pattern did not provide a clean sensitization signal. This finding is worth retaining because it prevents selective overstatement. It also helps preserve the article’s central discipline: not every anthocyanin-drug combination is automatically a sensitization model.

These exploratory contexts suggest that anthocyanin-rich preparations can modulate chemotherapy-associated stress responses, metastasis-related behavior, antioxidant-response pathways, and adaptation-linked gene programs. They do not yet provide the evidence package required for a central claim in this manuscript. A stronger claim would need a defined resistant breast cancer state, therapy-matched combination design, formal additivity or synergy testing, and a mechanistic bridge to NF-κB/EMT or a directly connected plasticity axis. These studies strengthen the biological rationale but do not form the basis for the central sensitization claim.

### Endocrine-resistant and CDK4/6-resistant disease as current evidence gaps

Endocrine-resistant and CDK4/6-resistant breast cancer currently represent evidence gaps rather than supported anthocyanin-sensitization settings. Clinically, both are highly important. Endocrine resistance remains a major problem in ER-positive breast cancer, and NF-κB has been linked to inflammatory survival signaling and endocrine therapy resistance [17]. CDK4/6 inhibitors have also changed the treatment landscape of HR-positive/HER2-negative advanced breast cancer, but resistance to endocrine therapy combined with CDK4/6 inhibition is now a central translational problem, with mechanisms involving cell-cycle bypass, RB-pathway disruption, PI3K/AKT/mTOR activation, growth-factor signaling, and broader adaptive rewiring [65, 66].

Clinical importance alone does not create an anthocyanin evidence block. The current literature does not yet show, in a focused way, that anthocyanins resensitize tamoxifen-resistant, aromatase inhibitor-resistant, fulvestrant-resistant, palbociclib-resistant, ribociclib-resistant, or abemaciclib-resistant breast cancer models. General anticancer, anti-inflammatory, anti-invasive, or chemopreventive data on anthocyanins are not enough for that claim [6].

The gap remains relevant because these resistant states are biologically compatible with the framework. Endocrine and CDK4/6 resistance often involves survival buffering, inflammatory signaling, growth-factor escape, cell-cycle plasticity, and transcriptional adaptation. In principle, these are settings in which anthocyanins could be tested rationally. But this remains an experimental direction, not a conclusion. Compared with trastuzumab-resistant HER2-positive models or taxane-related TNBC, the endocrine/CDK4/6 area lacks direct anthocyanin-combination studies, validated resistant derivatives, drug-interaction analysis, and therapy-response-linked NF-κB/EMT validation.

The conclusion should therefore remain conservative. Endocrine-resistant and CDK4/6-resistant breast cancer are clinically central and mechanistically plausible, but anthocyanin-specific sensitization evidence is currently too thin to shape the main argument of this article.

### Why these settings remain secondary in the present article

The settings discussed in this section define the boundary of the field. They show where anthocyanin-related evidence touches therapy response and where it remains short of state-specific sensitization. This boundary is necessary because, without it, the manuscript would drift toward a broad catalogue of anticancer phytochemical effects.

The strongest evidence remains concentrated in HER2-positive/trastuzumab-related models and taxane-related TNBC, where a treatment-relevant state, therapeutic backbone, measurable sensitization, and partial NF-κB/EMT or adjacent plasticity alignment can be connected more clearly. Cisplatin- and doxorubicin-related settings are more fragmented, and endocrine/CDK4/6-resistant disease remains largely undeveloped from an anthocyanin-specific perspective.

The secondary evidence is still useful. Vitis coignetiae anthocyanins provide an AKT/NF-κB-linked cisplatin potentiation signal in MCF-7 cells [60]. Dark sweet cherry anthocyanins add resistance-adjacent information in 4T1 models through effects on pulmonary metastasis, drug-pressure responses, and stress-adaptation pathways [58, 64]. Gardenblue blueberry anthocyanins and anthocyanin-based nanoformulations broaden the context through apoptosis-, stress-, antioxidant-response-, and formulation-related findings [61, 62]. However, most of these studies do not use validated acquired resistant breast cancer models, and they do not converge into a strong resistance-state framework. Current evidence, therefore, does not support clinical recommendations, broad dietary claims, or statements that anthocyanins reverse cisplatin, doxorubicin, endocrine, or CDK4/6 inhibitor resistance in breast cancer. These settings should remain secondary to the trastuzumab-resistant HER2-positive and taxane-related TNBC evidence blocks. Table 1 summarizes this state-specific distribution by separating direct sensitization signals from supportive resistance-adjacent biology and from clinically important but currently unproven resistance settings.

**Table 1:** Evidence hierarchy for anthocyanin-linked sensitization in therapy-resistant breast cancer

Resistant state/evidence block	Therapy context	Anthocyanin-related exposure	Core finding	Mechanistic alignment	Evidence level	Translational reading
HER2-positive/trastuzumab-resistant disease	Trastuzumab	Cyanidin-3-glucoside; peonidin-3-glucoside	Defined anthocyanins show activity in HER2-positive models, potentiate trastuzumab-relevant effects, and suppress trastuzumab-resistant HER2-positive derivatives in vitro and in vivo [7, 8, 40].	HER2/AKT/MAPK evidence is direct; NF-κB/EMT relevance is biologically plausible but not fully proven in the same anthocyanin-resistance design.	High preclinical	Priority block for further testing; best described as resistance-state activity with sensitization relevance, not definitive trastuzumab resensitization.
Paclitaxel-resistant TNBC	Paclitaxel	Bilberry-derived anthocyanidins	Anthocyanidins chemosensitize paclitaxel-resistant TNBC cells and xenografts; the combination lowered paclitaxel IC_50_ by nearly 20-fold in MDA-MB-231Tx cells, while monotherapies were ineffective in resistant xenografts [9].	Strongest NF-κB-linked block; invasion, metastasis and angiogenesis-related outputs support a resistance-plasticity interpretation.	High preclinical	Strongest TNBC signal; supports anthocyanidins as paclitaxel chemosensitizers in a defined resistant preclinical state.
Mesenchymal TNBC	Docetaxel	Delphinidin-3-O-glucoside; delphinidin-3-O-rutinoside; related anthocyani(di)ns	Trihydroxylated anthocyanins potentiated docetaxel in mesenchymal TNBC cells, with structure-dependent activity and dose-reduction signal [49].	Mesenchymal-state relevance is clear; causal NF-κB/EMT reversal remains incomplete.	Intermediate	Useful candidate-selection signal, especially for delphinidin derivatives; not yet validated resistance-reversal evidence.
Cisplatin low-sensitivity breast cancer models	Cisplatin	*Vitis coignetiae* anthocyanins; blueberry anthocyanins/formulations	Anthocyanin-containing preparations enhance cisplatin responsiveness mainly in low-sensitivity settings, especially MCF-7-based models [60].	AKT/NF-κB support is partial; EMT evidence is weak or absent.	Intermediate/low	Supportive chemosensitization evidence; should not be written as reversal of acquired cisplatin resistance.
Doxorubicin/drug-resistant 4T1-related settings	Doxorubicin or drug-pressure context	Dark sweet cherry anthocyanin-rich phenolics	Dark sweet cherry anthocyanins affect drug metabolism/transport, metastasis-related biology, and stress-response wiring in 4T1-based models [57, 58, 64].	Resistance-adjacent biology is present, including drug-efflux/metabolism and Nrf2–Keap1–p62-related stress adaptation; this is not an NF-κB/EMT core block.	Low/supportive	Useful adjacent evidence; keep outside the main sensitization claim.
Endocrine- and CDK4/6-resistant disease	Endocrine therapy; CDK4/6 inhibitors	No convincing anthocyanin-specific sensitization evidence	Clinically important resistance states, but no focused anthocyanin resensitization block is available for tamoxifen-, aromatase inhibitor-, fulvestrant-, palbociclib-, ribociclib- or abemaciclib-resistant breast cancer models [17, 66, 67].	Mechanistically plausible through inflammatory survival, growth-factor escape, cell-cycle rewiring and adaptive plasticity; experimentally unproven for anthocyanins.	Evidence gap	Mention as a rational future testing area only; do not upgrade into a supported anthocyanin-sensitization setting.

Evidence levels were assigned according to whether each evidence block combined a defined resistant or low-sensitivity breast cancer state, a therapy-relevant comparator or combination, a measurable sensitization or resistance-modifying endpoint, and mechanistic alignment with NF-κB, EMT, or an adjacent plasticity/stress-adaptation axis. **High preclinical** indicates direct state-linked evidence with therapy-combination or resistant-model support. **Intermediate** indicates treatment-relevant but mechanistically incomplete evidence. **Low/supportive** indicates resistance-adjacent biology without a strict resensitization design. **The evidence gap** indicates a clinically relevant resistance setting for which anthocyanin-specific sensitization data are currently insufficient. References are provided to keep the evidence trail transparent without overloading the main text. **Abbreviations: AKT**, protein kinase B; **CDK4/6**, cyclin-dependent kinases 4 and 6; **EMT**, epithelial–mesenchymal transition; **HER2**, human epidermal growth factor receptor 2; **IC**_**50**_, half-maximal inhibitory concentration; **MAPK**, mitogen-activated protein kinase; **NF-κB**, nuclear factor kappa B; **Nrf2**, nuclear factor erythroid 2-related factor 2; **PMID**, PubMed identifier; **TNBC**, triple-negative breast cancer

## Translational boundaries: chemistry, exposure realism, and minimum validation standards

The evidence summarized above supports a selective interpretation of anthocyanin-based sensitization in therapy-resistant breast cancer. The strongest signals remain confined to defined experimental contexts, particularly HER2-positive/trastuzumab-related models and taxane-related or mesenchymal TNBC. Before these signals can be moved closer to translation, three issues must be handled more rigorously: chemical identity, exposure realism, and validation of the resistant state. Without these filters, even biologically interesting findings risk being overread as clinically meaningful adjunct evidence.

### Anthocyanins versus anthocyanidins: why the distinction matters

Although this manuscript focuses on anthocyanins, the chemical scope has to remain precise. Anthocyanins are glycosylated forms of anthocyanidins, and structural differences such as glycosylation, hydroxylation, methoxylation, and acylation can influence stability, polarity, metabolism, cellular uptake, and biological activity. This distinction directly affects the interpretation of sensitization data, as emphasized in chemical and nutritional reviews by Khoo et al. and Mattioli et al. [68, 69].

This distinction is particularly relevant in TNBC, where one of the strongest paclitaxel-resistance signals comes from bilberry-derived anthocyanidins rather than from a single purified anthocyanin glycoside; in paclitaxel-resistant MDA-MB-231Tx cells, anthocyanidins chemosensitized TNBC to paclitaxel through NF-κB-linked mechanisms [9]. The term “anthocyanin-based” is therefore best reserved for mixtures, enriched fractions, or chemically unresolved preparations. Where individual compounds are tested, such as cyanidin-3-glucoside, peonidin-3-glucoside, delphinidin-3-O-glucoside, or delphinidin-3-O-rutinoside, they should be named explicitly. This avoids implying a uniform class effect and keeps the chemical claim aligned with the actual evidence.

### Pure compounds, defined mixtures, and chemically vague extracts

Anthocyanin evidence differs substantially in translational weight. Pure compounds provide the cleanest mechanistic interpretation, because the relationship between structure, model, and phenotype is more traceable. Chemically defined enriched mixtures are also valuable, especially when they resemble source-derived preparations more closely, but they require compositional profiling and batch control. Poorly characterized extracts remain supportive unless their anthocyanin fraction is analytically defined, because inadequate phytochemical characterization limits reproducibility and weakens pharmacological interpretation [70].

This hierarchy matters because “anthocyanin-rich” is not a pharmacological identity. Berry extracts may contain anthocyanins, anthocyanidins, proanthocyanidins, flavonols, phenolic acids, sugars, organic acids, and other matrix-dependent constituents. Reviews on anthocyanin bioavailability and whole-food versus extract exposure make clear that matrix, processing, metabolism, and chemical form can substantially modify biological interpretation [71, 72]. Therefore, a purified cyanidin glycoside, a bilberry anthocyanidin fraction, and a broad berry extract should not be treated as equivalent evidence in a resistance-sensitization framework.

### Bioavailability, stability, metabolism, and formulation barriers

The main translational problem is exposure plausibility. Anthocyanins show biological activity in experimental systems, but the tested concentrations and chemical forms must be judged against what resistant tumor cells could plausibly encounter in vivo. Anthocyanins are chemically sensitive, and their stability is influenced by pH, temperature, oxygen, light, enzymes, co-pigmentation, and matrix composition, as summarized by Enaru et al. [73].

Human exposure is also metabolically complex. Parent anthocyanins may appear at low concentrations, while conjugates, degradation products, and microbiota-derived metabolites can contribute substantially to systemic exposure. De Ferrars et al. and Kay et al. show why anthocyanin pharmacokinetics should not be reduced to the parent compound alone [72, 74]. This directly limits the strength of cell-culture claims: a micromolar effect of a parent anthocyanin is useful mechanistically, but it is not automatically translatable to breast-tumor exposure after oral intake.

Formulation may partly address this limitation. Nanoemulsion and nanoliposome strategies have been explored to improve anthocyanin stability, bioavailability, and biological activity, as reviewed by Chen and Inbaraj [75]. Formulation-supported exposure should therefore be separated from food-achievable exposure. If sensitization depends on a delivery system, the conclusion belongs to formulation-dependent adjunct development rather than dietary recommendation.

### What should count as exposure realism in future sensitization studies

Future studies need to specify whether the tested exposure is diet-achievable, supplement-like, metabolite-relevant, or formulation-dependent. This does not require full clinical pharmacokinetics in every preclinical paper, but it does require a serious exposure argument. The distinction between in vitro potency and plausible tumor exposure should be visible, not hidden in the discussion.

A stronger design would also be schedule-aware. Pretreatment before chemotherapy, concurrent exposure, and post-treatment exposure may answer different biological questions. The same applies to trastuzumab-related studies: enhanced activity in a HER2-positive model is not identical to reversal of established trastuzumab resistance. Combination experiments should therefore include standard therapy alone, anthocyanin or anthocyanidin alone, the combination, and formal interaction analysis. Chou’s work on drug-combination assessment remains a useful methodological anchor for separating additivity, synergy, and parallel cytotoxicity [41].

Metabolite biology should also be considered. Human pharmacokinetic data with ^13 C-labelled cyanidin-3-glucoside show that systemic exposure reflects a structurally diverse pool of degradation products and metabolites, not only the parent compound [74, 76]. Parent-compound activity should therefore be treated as mechanistic evidence rather than as translationally persuasive evidence unless metabolite relevance is addressed. For a 3PM-oriented framework, biomarker-guided prioritization has limited value unless the proposed adjunct can plausibly reach the relevant biological compartment.

### Minimum validation standards for future studies

“Sensitization” requires stricter evidence than ordinary growth inhibition. At minimum, future work needs resistant-state confirmation, a clinically relevant therapy backbone, combination testing, mechanistic alignment between claim and readout, and a basic pharmacologic or formulation rationale.

Resistant models should be documented, not assumed. Future studies should report the parental comparator, resistance-generation method or provenance, resistance index, stability of the phenotype, and pathway or marker features relevant to the claimed state. McDermott et al. outline why the development and maintenance of resistant cancer cell lines can materially affect interpretation [77].

Mechanistic claims require the same level of discipline. If NF-κB is invoked, studies should measure proximal NF-κB activity or transcriptional output, not only downstream changes. If EMT is invoked, migration or invasion alone is insufficient; epithelial and mesenchymal markers should be assessed, ideally together with recognition of partial or hybrid EMT states. If the claim is drug re-sensitization, these molecular changes should track with restored therapy response, not merely with nonspecific cytotoxicity.

**Fig. 1:**
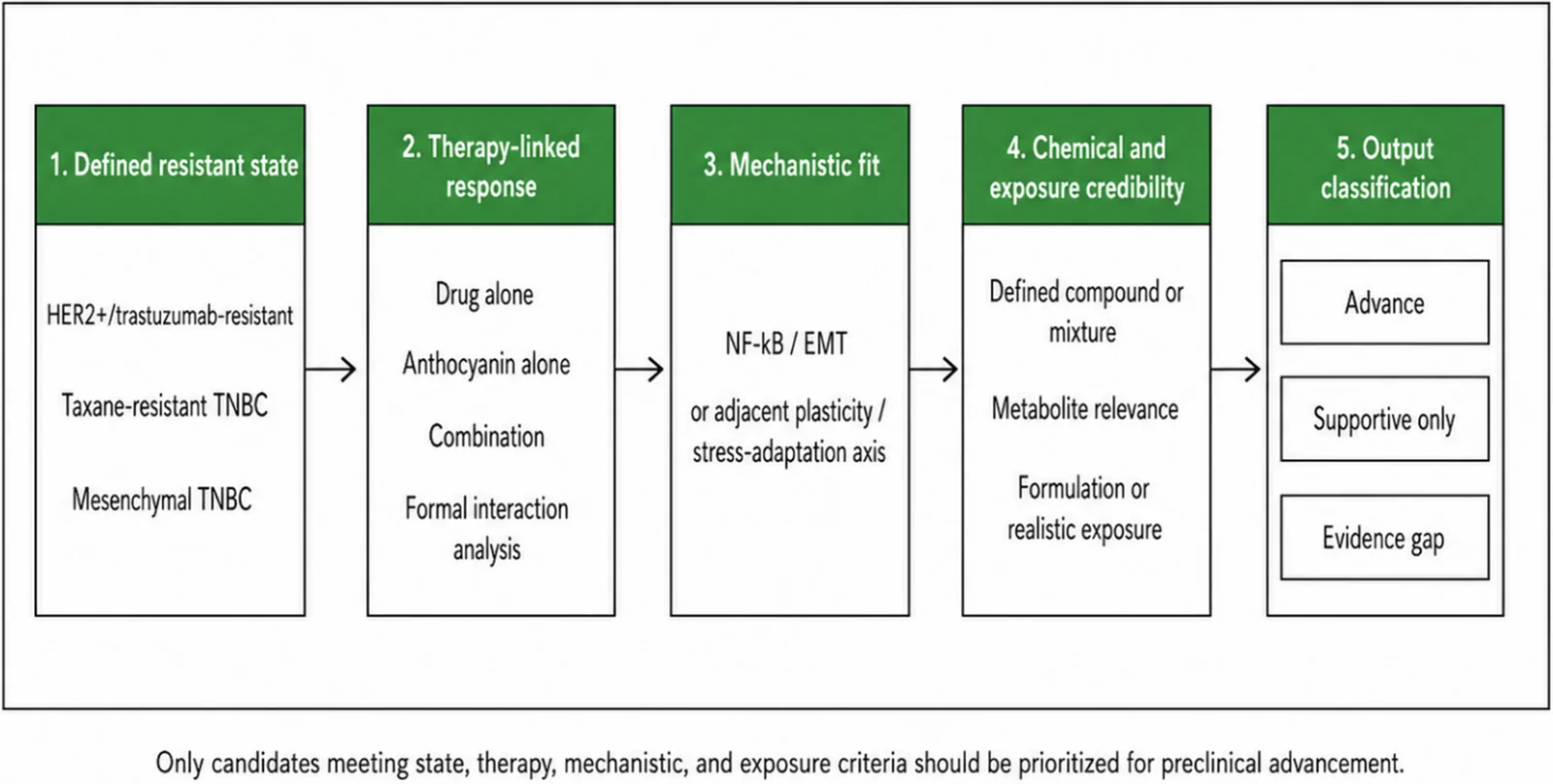
Decision framework for anthocyanin/anthocyanidin prioritization in therapy-resistant breast cancer; The scheme outlines a stepwise decision process for evaluating anthocyanin- or anthocyanidin-based sensitization evidence. Candidate interventions are first anchored in a defined resistant breast cancer state and then tested in a therapy-linked design that includes drug-alone, anthocyanin-alone, and combination arms. Mechanistic interpretation relies on NF-κB/EMT or adjacent plasticity/stress-adaptation readouts, together with chemical identity and exposure credibility, before classification as advanced, supportive, or unresolved. Abbreviations: EMT, epithelial–mesenchymal transition; HER2, human epidermal growth factor receptor 2; NF-κB, nuclear factor kappa B; TNBC, triple-negative breast cancer

Under these standards, the field remains biologically plausible but not clinically actionable. The strongest evidence supports biomarker-guided preclinical prioritization of selected anthocyanin or anthocyanidin candidates in defined resistant states. It does not support broad claims that anthocyanins overcome breast cancer therapy resistance or justify clinical supplementation recommendations. The next step is chemically defined, exposure-aware, resistance-validated combination testing. Figure 1 translates the evidence hierarchy into a decision framework, separating candidates that merit further preclinical testing from supportive mechanistic observations and from currently unproven resistance settings.

## 3PM-guided conceptual innovation and improved individual outcomes

### Patient phenotyping and stratification as the essential pillar

#### Patient case analysis 1: 3PM-guided rehabilitation after treated TNBC and targeted prevention against metastatic disease

A female premenopausal patient, 32 years old with BMI = 20, was diagnosed with and treated for the TNBC. The patient is currently considered cured but suffers from chronic fatigue and wishes to undergo professionally-guided rehabilitation in order to reduce risks of metastatic disease in the future. The patient does not demonstrate any genetic predisposition to BC, nor cases of BC in the family. The evidence-based individualised rehabilitation program (see more details dedicated to the topic [78] is recommended to consider following topic-relevant phenotype of the patient:

- Systemic peripheral ischemia-reperfusion resulting from disturbed peripheral microcirculation
- Stress vulnerability and chronification
- Altered termoregulation
- Altered drug sensitivity & shifted MDR transporter activation patterns
- Mitochondrial homeostasis as the natural biosensor of systemic effects.

All the above listed patterns are linked to the functional involvement of pathways related to the by NF-kappa B signaling [19, 79–86].

#### Patient case analysis 2: young TNBC patients with pre-metastatic niches as a specific phenotype for cost-effective screening programs

For a female premenopausal patient aged 28 years, a tumor below 1 cm of size was diagnosed in the breast spreading multiple-site metastases detected in the bone and liver. The patient clearly demonstrates psycho-somatic features characteristic for the sympathetic overdrive syndrome (SOP) [87–89] including dominant sympathetic over-excitation, chronification of stress, systemic inflammation, impaired wound healing, chronic fatigue, mitochondrial burnout characterized by significantly shifted mitochondrial homeostasis, and SOP-specific highly aggressive metastatic TNBC. To this end, pathomechanisms which underlie the formation of pre-metastatic niches in the Flammer Syndrome phenotype/SOP carriers are sufficiently detailed in the topic-dedicated scientific literature [90, 91]. Chronic sympatho-excitation is defined as the sustained dominance of sympathetic over parasympathetic tone, which is systemically measurable. Despite a certain level of genetic predisposition to the SOP, the key pathways are epigenetically regulated presenting therefore a promising target for the cost-effective holistic approach [90]. Contextually, the 3PM-guidlines strongly recommend an application of the

- SOP scoring and health risk assessment in primary and secondary care
- SOP-associated biomarker-panels relevant for the malignant transformation and formation of pre-metastatic niches
- individualised patient profiling for the tailored treatment algorithms and digital health monitoring.

### Predictive relevance: Which tumors might plausibly fit this approach?

The strongest 3PM relevance of this field is predictive. Anthocyanin-based adjunct strategies are most plausible in tumors that already show a resistance phenotype compatible with the proposed mechanism: NF-κB activation, EMT features, mesenchymal drift, inflammatory signaling, invasive behavior, or related plasticity markers. This is consistent with the broader logic of breast cancer precision medicine, where treatment selection depends on predictive biomarkers rather than on tumor type alone, as discussed by Rodrigues-Ferreira and Nahmias in their review of breast cancer predictive biomarkers [13].

Within the evidence reviewed here, two settings are currently the most defensible: HER2-positive/trastuzumab-related resistance and taxane-related or mesenchymal TNBC. The TNBC signal is particularly relevant because bilberry-derived anthocyanidins chemosensitized paclitaxel-resistant TNBC through NF-κB-linked mechanisms in preclinical models, while mesenchymal TNBC data support taxane potentiation in a plasticity-enriched context. The predictive question is whether a resistant tumor state carries pathway features that make an anthocyanin or anthocyanidin adjunct biologically plausible [32].

### Preventive relevance as biological context

Anthocyanins have a substantial prevention-oriented literature, including antioxidant, anti-inflammatory, anti-invasive, and chemopreventive activity in breast cancer models, as summarized by Li et al. and supported by systematic TNBC evidence showing effects on migration, invasion, AKT/mTOR signaling, EMT-related markers, and apoptosis. This literature remains secondary in the present manuscript. Prevention is relevant as biological context, whereas the main translational issue is whether chemically defined anthocyanin or anthocyanidin candidates can support drug response in defined resistant states. This distinction is consistent with a 3PM-oriented view of breast cancer in which pathway activity, molecular profiling, plasticity, and therapeutic resistance—not broad nutraceutical exposure—should guide translational prioritization. Merging state-specific sensitization with general dietary prevention would blur the manuscript’s main contribution [6, 92, 93].

### Personalized relevance: phenotype-matched adjunct hypotheses

From a personalized-medicine perspective, the value of this evidence is hypothesis-generating. It may help define phenotype-matched adjunct strategies for future testing. In practical terms, this means selecting a resistant state, defining its dominant survival or plasticity features, matching those features to a chemically specified anthocyanin or anthocyanidin intervention, and then testing the combination against the relevant therapy backbone. This interpretation is consistent with a 3PM view of flavonoids as modulators of breast cancer cell plasticity and therapeutic resistance, where pathway context and resistance phenotype should guide candidate prioritization rather than broad nutraceutical claims. Biomarker- and metabolomics-based stratification in breast cancer patients further supports this logic, because personalized adjunct strategies require measurable biological context before translational claims can be made [94, 95].

This approach is aligned with the biomarker logic of 3PM, where measurable traits are used to predict therapeutic response or risk of treatment failure rather than to support generic intervention claims. Sacco and Grech describe predictive biomarkers as measurable features that may identify patients likely to benefit from treatment or experience adverse effects, which fits the logic needed here [14]. For anthocyanins, NF-κB-high, EMT-enriched, inflammatory, mesenchymal, or taxane-resistant tumors should be treated as testable hypotheses, not as patient groups for immediate intervention.

### What cannot yet be claimed

Current evidence does not justify a clinical recommendation. Anthocyanins, anthocyanidins, berry extracts, and anthocyanin-rich foods should not be presented as treatment adjuncts for patients with therapy-resistant breast cancer. The evidence also does not support broad claims that anthocyanins overcome trastuzumab, taxane, cisplatin, endocrine, or CDK4/6 inhibitor resistance in clinical disease [78].

Within a 3PM framework, the defensible position is limited to biomarker-guided preclinical hypothesis generation in resistant states marked by NF-κB activation, EMT-associated plasticity, inflammatory survival signaling, or taxane-linked adaptation. Stronger claims require chemically defined interventions, validated resistant models, formal interaction testing, matched mechanistic readouts, exposure-realistic dosing, and in vivo systems that better reflect tumor heterogeneity and treatment pressure [32, 89, 94, 96].

## Conclusions

Anthocyanins and anthocyanidins are most credibly positioned as candidate adjuncts for selected resistant states in which inflammatory survival signaling, EMT-associated plasticity, mesenchymal behavior, or NF-κB-linked therapy tolerance is biologically relevant.

The most convincing evidence currently comes from HER2-positive/trastuzumab-related models and from taxane-related or mesenchymal TNBC. The most coherent mechanistic theme is not simple cytotoxicity, but interference with resistance-associated plasticity, especially where NF-κB and EMT-linked behavior contribute to reduced drug response. This interpretation is consistent with systematic evidence in TNBC that anthocyanin effects should not be generalized across models or contexts.

Stronger translational claims require greater chemical precision, exposure-aware design, and a more rigorous definition of resistance. Current preclinical evidence supports anthocyanins as plausible adjunct sensitizers in selected therapy-resistant breast cancer states, particularly trastuzumab-resistant HER2-positive disease and taxane-related or mesenchymal TNBC. The field, however, remains chemically heterogeneous and translationally constrained.

## Data Availability

No datasets were generated or analysed during the current study.

## Declarations

### Generative AI and AI-assisted technologies in the manuscript preparation process

During the preparation of this work, the authors used AI-assisted language-editing tools to support grammar, readability, and phrasing. After using these tools, the authors reviewed and edited the manuscript as needed and take full responsibility for the content of the published article. All scholarly decisions, including literature selection, evidence grading, interpretation, conclusions, references, and final content, were made and approved by the authors. AI-assisted tools were used only for language editing and readability refinement, not for literature selection, reference generation, evidence grading, figure generation, data interpretation, or scientific conclusions.

## Abbreviations

3PM/PPPM: predictive, preventive, and personalized medicine
AI: artificial intelligence
AKT: protein kinase B
Bax: Bcl-2-associated X protein
BC: breast cancer
Bcl-2: B-cell lymphoma 2
BMI: body mass index
CDK4/6: cyclin-dependent kinases 4 and 6
COX-2: cyclooxygenase-2
c-Myc: cellular myelocytomatosis oncogene product
c-Src: cellular Src tyrosine kinase
EGFR: epidermal growth factor receptor
EMT: epithelial–mesenchymal transition
EPMA: European Association for Predictive, Preventive and Personalized Medicine
ER: estrogen receptor
ERα36: estrogen receptor alpha 36-kDa variant
FAK: focal adhesion kinase
HER2: human epidermal growth factor receptor 2
HR: hormone receptor
IC₅₀: half-maximal inhibitory concentration
ICAM-1: intercellular adhesion molecule 1
IKK: IκB kinase
IL-6: interleukin-6
IκBα: inhibitor of nuclear factor κB alpha
JAK: Janus kinase
Keap1: Kelch-like ECH-associated protein 1
KLF4: Krüppel-like factor 4
MAPK: mitogen-activated protein kinase
MDR: multidrug resistance
MMPs: matrix metalloproteinases
mTOR: mechanistic target of rapamycin
NF-κB: nuclear factor kappa B
Nrf2: nuclear factor erythroid 2-related factor 2
p53: tumor protein p53
p62: sequestosome 1
p65: RelA/p65 subunit of NF-κB
p130Cas: p130 Crk-associated substrate
p-IκB: phosphorylated inhibitor of nuclear factor κB
p-NF-κB: phosphorylated nuclear factor κB
PARP-1: poly(ADP-ribose) polymerase 1
PI3K: phosphoinositide 3-kinase
PMID: PubMed identifier
PTEN: phosphatase and tensin homolog
RB: retinoblastoma protein
ROS: reactive oxygen species
SANRA: Scale for the Assessment of Narrative Review Articles
Sirt1: sirtuin 1
SLUG: Snail family transcriptional repressor 2
SNAIL: Snail family transcriptional repressor
SOP: sympathetic overdrive syndrome
STAT: signal transducer and activator of transcription
STAT3: signal transducer and activator of transcription 3
TGF-β: transforming growth factor beta
TNBC: triple-negative breast cancer
TNF-α: tumor necrosis factor alpha
TWIST: Twist family transcription factor
VEGA: Scientific Grant Agency of the Slovak Republic
VEGF: vascular endothelial growth factor
XIAP: X-linked inhibitor of apoptosis protein
ZEB: zinc finger E-box-binding homeobox
ZO-1: zonula occludens-1
Model and cell: line designations
4T1: murine mammary carcinoma cell line/model
4T1-DR: drug-resistant 4T1 cell model
HeLa: human cervical carcinoma cell line
HepG2: human hepatocellular carcinoma cell line
MCF-7: ER-positive human breast cancer cell line
MDA-MB-231: human triple-negative breast cancer cell line
MDA-MB-231Tx: paclitaxel-resistant MDA-MB-231 cell line
MDA-MB-453: HER2-positive human breast cancer cell line
